# Persistently high prevalence of primary resistance and multidrug resistance of tuberculosis in Heilongjiang Province, China

**DOI:** 10.1186/s12879-016-1848-9

**Published:** 2016-09-27

**Authors:** Di Li, Jing-Li Wang, Bin-Ying Ji, Jia-Yi Cui, Xin-Ling Pan, Chang-Long Fan, Chang-Xia Shao, Li-Na Zhao, Yuan-Ping Ma, Liu-Zhuo Zhang, Chun-Lei Zhang, Cai-Bo Dong, Toshio Hattori, Hong Ling

**Affiliations:** 1 Department of Microbiology; Wu Lien-Teh Institute, Harbin Medical University, Harbin, China; 2 Heilongjiang Provincial Key Laboratory of Infection and Immunity, Harbin, China; 3 Department of Parasitology, Harbin Medical University; Key Laboratory of Pathogen Biology, Harbin, China; 4 Harbin Chest Hospital, Harbin, China; 5 Shen Zhen Protection and Treatment Center for Occupational Diseases, Shenzhen, China; 6 Division of Disaster-related Infectious Diseases, International Research Institute of Disaster Science, Tohoku University, Sendai, Japan

**Keywords:** *Mycobacterium tuberculosis*, Primary resistance, Multi-drug resistance, Risk factors

## Abstract

**Background:**

The spread of multidrug-resistant tuberculosis (MDR-TB) *Mycobacterium tuberculosis* (*M. tuberculosis*) strains has been a big challenge to the TB control and prevention in China. Knowledge about patterns of drug resistance in TB high-burden areas of China is crucial to develop appropriate control strategies. We conducted a comprehensive investigation of the resistance pattern of *M. tuberculosis* in Heilongjiang Province.

**Methods:**

1427 *M. tuberculosis* clinical strains were isolated from pulmonary TB patients hospitalized between 2007 and 2012. The susceptibility of the isolates to the first-line anti-TB drugs and the resistance of MDR *M. tuberculosis* to fluoroquinolones were examined. We also performed a statistical analysis to identify the correlated risk factors for high burden of MDR-TB.

**Results:**

The global resistance rates of 2007–2012 to the first-line drugs and MDR were 57.0 and 22.8 %, respectively. Notably, the primary MDR-TB and pan-resistance rates were as high as 13.6 and 5.0 %, respectively. Of MDR *M. tuberculosis* isolates (2009), approximately 13 % were not susceptible to any of the fluoroquinolones tested. Being age of 35 to 54, high re-treatment proportion, the presence of cavity lesion, and high proportion of shorter hospitalization are correlated with the development of MDR-TB.

**Conclusions:**

The high prevalence of drug resistant, MDR-TB, and fluoroquinolone-resistant MDR-TB is a big concern for TB control. More importantly, in order to control the development of MDR-TB effectively, we need to pay more attention to the primary resistance. Targeting reducing the prevalence of the risk factors may lead to better TB control in China.

## Background

The spread of drug-resistant and multidrug-resistant (MDR) tuberculosis (TB) is a severe global health issue. In recent years, both the incidence and prevalence of TB in China have steadily declined [1]. The World Health Organization (WHO) indicates that the MDR rates in China (5.7 % in new cases and 26 % in previously treated cases, respectively) have become lower than in other countries among the 27 high MDR-TB burden countries. However, the rates remain higher than the global average rates of 3.6 and 20 % for new and previously treated cases, respectively. China is ranked second in the number of MDR-TB cases after India because of China’s large population. Moreover, the global extensively drug-resistant (XDR)-TB prevalence is increasing, with 9.6 % MDR-TB cases in 2012 compared to 5.4 % in 2007. We estimate an increasing XDR-TB prevalence in China, with 8 % from MDR-TB cases, because this rate was higher than the worldwide level in 2007 [1]. The global spread of XDR-TB has led to new challenges for the prevention and control of tuberculosis.

The epidemic trends of drug resistant-TB in different geographic areas of China vary greatly, and recently, the spread of TB in China has been caused by active domestic migration, resulting in a significant public health issue. Heilongjiang Province is located in Northeast China, a region in which the prevalence rates of both TB and drug-resistant TB are higher than the average in China [2–4]. The rates of any resistance to first-line drugs were 38.9 and 36.2 % for new cases and 70.9 and 67.7 % for re-treated cases in Heilongjiang Province in 2002 and 2004, respectively, based on data from population-based drug resistance investigations [2–4].

It is important to identify the causes for the high prevalence of drug-resistance TB. The risks associated with drug resistant- and MDR-TB include a history of retreatment using anti-mycobacterial drugs, having cavities, Beijing genotype epidemic, low socio-economic status, age and DOTS (directly observed treatment short course) implementation. However, the factors vary depending on the study [1, 5–8].

Moreover, we would like to determine whether the prevalence of fluoroquinolone resistant TB in Heilongjiang Province is also high. MDR-TB is a risk factor for the resistance of *M. tuberculosis* to fluoroquinolones [9, 10]. In China, fluoroquinolones are the most frequently used antibiotics for the treatment of a variety of infectious diseases [11–14]. XDR-TB arises when MDR-TB strains acquire resistance to any fluoroquinolone, and previous treatment using second-line drugs, including fluoroquinolones, is a significant risk factor for XDR-TB [15, 16].

In the present study, we analyzed the resistance trends of *M. tuberculosi*s clinical isolates from 2007 to 2012 to first-line drugs and fluoroquinolones and the risk factors in Heilongjiang Province, an area with the highest tuberculosis disease and drug resistance burden in China. Elucidating the locally significant risk factors for the high prevalence of TB is important to control the spread of TB and drug resistant TB in this area and other regions of China.

## Methods

### Mycobacterial specimen and data collection

All the data we collected including patient medical records are available in Harbin Chest Hospital. A total of 1427 isolates from 1427 patients, who were diagnosed with TB at the Harbin Chest Hospital from May 2007 and July 2012 and from various regions of Heilongjiang Province, were included. The patients were HIV-1 negative. The patient information of gender, age, TB treatment history, and the presence of cavity on chest radiographs was from hospital medical records. Ethical clearance and approval for this study was provided by the Institutional Research Board of the University of Harbin Medical University (Ethics Reference No.: HMUIRB20160001).

### Mycobacterial culture and drug susceptibility testing (DST) to first-line drugs

Mycobacterial cultures were obtained from clinical specimens after incubation in a BACTEC Mycobacterium Growth Indicator Tube (MGIT) 960 Automated System (BD Diagnostic Systems, Franklin Lakes, NJ, USA). Primary identification was performed using the Ziehl-Neelsen staining method and microscopy. The tests of inhibition by P-nitrobenzoic acid and 2-Thiophenecarboxylic acid hydrazide were used to differentiate *M. tuberculosis* from other *Mycobacterium spp*. DST to first-line drugs was performed using MGIT 960 SIRE Kit. Strict controls including growth control (*M. tuberculosis* H37Rv growth control in drug-free tube) and the negative control (H37Rv in the presence of each drug) were used according to the instruction of MGIT 960 SIRE Kit.

### The resistance of MDR *M. tuberculosis* to fluoroquinolones

Resistance of some MDR isolates from 2009 to 2012 to FQs was examined in the Hospital as clinical departments suggested. The susceptibility of the MDR strains isolated in 2009 (*n* = 52, recovered from the frozen stock) to FQs including levofloxacin (Sigma), sparfloxacin (Sigma), moxifloxacin (Hubei Saibo Pharmaceutical company, Wuhan, China), gemifloxacin (Hubei Saibo Pharmaceutical company, Wuhan, China), and gatifloxacin (National Institutes for Food and Drug Control, Beijing, China) was examined by minimun inhibition concentrations (MIC) assay. The test was performed using the standard microdilution method as described previously [17]. Initial concentration was 16 mg/L for both levofloxacin and sparfloxacin, and 8 mg/L for moxifloxacin, gemifloxacin, and gatifloxacin. Seven serial two-fold dilutions of each drug in Middlebrook 7H9 were performed [18–20]. Each well was inoculated with a 0.03 McFarland mycobacterial suspension (final inoculums was 2.85 × 10^5^ CFU/mL). Two wells without drugs were inoculated with H37Rv as a growth control. All peripheral wells of the plates were filled with sterile distilled water. The plates were incubated at 37 °C for 16 to 18 days. For each drug, the lowest concentration that displayed no visible turbidity was defined as the MIC. Three independent assays were performed.

The susceptibility of MDR isolates from 2010 to 2012 to levofloxacin was determined by using absolute concentration method on solid Lowenstein-Jensen medium (Hangzhou Genesis Biodetection & Biocontrol Ltd) upon National Guideline. The concentration of 2 mg/L was set as the breakpoint [21].

### DNA extraction, PCR-based identification of *M. tuberculosis*, and Beijing genotype determination

Bacterial DNA was extracted after the inactivation of mycobacterial isolates in 70 % ethanol for 2 h [22]. DNA was extracted using lysozyme and the phenol-chloroform method [23]. Molecular identification of *M. tuberculosis* with the PCR amplifications of the genes including 16S rRNA, Rv0577, Rv2073c, and Rv3120 was performed as previously described [24, 25]. *M. tuberculosis* strains have positive amplifications of the four genes. *M. microti* and *M. tuberculosis* H37Rv was used as reference strains.

To identify the Beijing genotype of the isolates, RD105 deletion PCR was performed as described previously [24].

### Statistical analysis

The odds ratio was used to evaluate the univariate and multivariate risk factors associated with MDR-TB and drug resistant- (not MDR) TB. The following variables were included in the analysis: the patient’s gender, age, TB treatment history, presence of a cavity on chest radiograph, hospitalization time and Beijing genotype. All of the variables were initially included in the model, and the forward method was used to select the final variables. The statistical interaction between relevant variables was assessed. All statistical analyses were performed using SPSS version 17.0 (SPSS Inc., Chicago, IL, USA).

## Results

### Sample and data collection

Of the 1427 patients with pulmonary tuberculosis identified from May 2007 through July 2012, 918 (64.3 %) and 509 (35.7 %) were new and re-treated cases, respectively (Table 1). The medical records were available for all cases. Chest radiographs indicating the presence or absence of cavity were available for 1254 cases. Three hundred isolates from May 2007 through September 2009 were re-confirmed by PCR-based identification and were further examined to identify the Beijing family genotype and phylogeny based on the VNTR genotypes. Fifty two re-cultured available MDR *M. tuberculosis* isolates from 2009 were examined for sensitivity to fluoroquinolones (MIC). The susceptibility of MDR isolates from 2010 to 2012 to levofloxacin was available from routine clinical drug resistance examinations in hospital.

**Table 1:** Susceptibility of the *M .tuberculosis* isolated in Heilongjiang Province to the first-line anti-tuberculosis drugs

Characteristics	2007		2008		2009		2010		2011		2012		Total	
	No.	%	No.	%	No.	%	No.	%	No.	%	No.	%	No.	%
All	198		348		275		204		205		197		1427	
Any drug resistance	110	55.6	209	60.1	189	68.7	121	59.3	89	43.4	96	48.7	814	57.0
RPF or INH^a,b^	42	21.2	77	22.1	72	26.2	55	27.0	42	20.5	40	20.3	328	23.0
SM	72	36.4	152	43.7	125	45.5	86	42.2	63	30.7	63	32.0	561	39.3
EMB	47	23.7	74	21.3	75	27.3	16	7.8	17	8.3	18	9.1	247	17.3
MDR	44	22.2	90	25.9	81	29.5	39	19.1	33	16.1	38	19.3	325	22.8
Pan-resistance^c^	21	10.6	49	14.1	37	13.5	10	4.9	12	5.9	11	5.6	140	9.8
From new cases	126		218		171		143		136		124		918	
Any drug resistance	62	49.2	113	51.8	103	60.2	74	51.7	46	33.8	49	39.5	447	48.7
RPF or INH	24	19.0	43	19.7	46	26.9	37	25.9	24	17.6	23	18.5	197	21.5
SM	42	33.3	84	38.5	67	39.2	53	37.1	32	23.5	31	25.0	309	33.7
EMB	19	15.1	32	14.7	38	22.2	9	6.3	6	4.4	5	4.0	109	11.9
MDR	20	15.9	36	16.5	29	17.0	18	12.6	11	8.1	11	8.9	125	13.6
Pan-resistance	5	4.0	17	7.8	14	8.2	4	2.8	4	2.9	2	1.6	46	5.0
From re-treated cases	72		130		104		61		69		73		509	
Any drug resistance	48	66.7	96	73.8	86	82.7	47	77.0	43	62.3	47	64.4	367	72.1
RPF or INH	18	25.0	34	26.2	26	25.0	18	29.5	18	26.1	17	23.3	131	25.7
SM	30	41.7	68	52.3	58	55.8	33	54.1	31	44.9	32	43.8	252	49.5
EMB	28	38.9	42	32.3	37	35.6	7	11.5	11	15.9	13	17.8	138	27.1
MDR	24	33.3	54	41.5	52	50.0	21	34.4	22	31.9	27	37.0	200	39.3
Pan-resistance	16	22.2	32	24.6	23	22.1	6	9.8	8	11.6	9	12.3	94	18.5

^a^ *INH* isoniazid, *RFP* rifampicin, *MDR* multidrug-resistance, *SM* streptomycin, *EMB* ethambutol

^b^The isolates were resistant to RPF or INH but not both

^c^The isolates were resistant to all the four first-line drug

### High prevalence of drug-resistant TB in Heilongjiang Province from 2007 to 2012

Overall, the average resistance rate of TB to any first-line drugs from 2007 to 2012 was 57.0 % (Table 1). The drug resistance rate among new case and re-treated case were 48.7 and 72.1 % respectively. The MDR-TB rate was 22.8 %. Of the MDR cases, 13.6 and 39.3 % were new and re-treated cases, respectively. The highest prevalence of resistance to the first-line drugs and MDR from either the new or retreated cases occurred in 2009 and the lowest occurred in 2011 (Table 1).

There was also a high prevalence of primary resistance (Table 1). Of the cases resistant to any first-line drug and MDR-TB cases, approximately 55 and 39 %, respectively, were new cases. From 2007 to 2012, the yearly resistance to any first-line drug among new cases, was 49.2, 51.8, 60.2, 51.7, 33.8, and 39.5 %, respectively. The MDR rates were 15.9, 16.5, 17.0, 12.6, 8.1, and 8.9 %, respectively. Notably, 5.0 % of the pan-resistant cases (resistant to all 4 first-line drugs) were detected in the new cases.

Importantly, the resistance of the re-treated cases was significant (Table 1). Of the cases resistant to any of the first-line drugs and MDR-TB cases, approximately 45.1 and 61.5 %, respectively, were re-treated cases. From 2007 to 2012, the resistance rates of the re-treated cases to any first-line drug were 66.7, 73.8, 82.7, 77.0, 62.3, and 64.4 %, respectively. The annual rates of MDR were 33.3, 41.5, 50.0, 34.4, 31.9, and 37 %, respectively. Of the re-treated cases, 18.5 % were pan-resistant.

### The resistance of MDR *M. tuberculosis* isolates to fluoroquinolones

Because the resistance rate to the first-line drugs in 2009 was the highest, resistance to fluoroquinolones of some MDR isolates (MIC) was conducted upon suggestion of clinical departments in strengthening the efficacy of treatment (Table 2). The critical concentration for levofloxacin is 2.0 mg/L, as per the World Health Organization, and was used to categorize the isolates as susceptible or non-susceptible. Because there are no recommended critical concentrations for sparfloxacin, moxifloxacin, gatifloxacin and gemifloxacin, we further analyzed the proportion of isolates with MICs equal to and higher than 2 mg/L, based on the serum concentrations of the drugs [20], and they were defined as non-sensitive to a drug.

**Table 2:** Susceptibilities of MDR *M. tuberculosis* isolates of 2009 to fluoroquinolones^a^

MIC (mg/L)	Levofloxacin		Moxifloxacin		Sparfloxacin		Gemifloxacin		Gatifloxacin	
	No.	%	No.	%	No.	%	No.	%	No.	%
≤0.125							2	3.8	1	1.9
≤0.25	7	13.4	5	9.6	14	26.9	7	13.5	8	15.4
0.50	10	19.2	18	34.6	9	17.3	14	26.9	15	28.8
1 mg/L	15	28.8	15	28.8	10	19.2	9	17.3	7	13.5
2.00	8	15.4	9	17.3	8	15.4	14	26.9	12	23.1
4.00	8	15.4	3	5.8	7	13.5	6	11.5	7	13.5
8.00	4	7.7	2	3.8	3	5.8			2	3.8
16.00					1	1.9				
MIC ≥ 2 mg/L	20	38.5	14	26.9	19	36.5	20	38.4	21	40.4
MIC50^b^	1.0		1.0		1.0		1.0		1.0	

^a^ *MDR* multidrug resistance, *MIC* minimum inhibitory concentration

^b^MIC_50_, minimum inhibitory concentration required to inhibit the growth of 50 % of examining clinical isolates

Of the MDR isolates, 36.5, 26.9, 38.4, and 40.4 % were not susceptible to sparfloxacin, moxifloxacin, gemifloxacin and gatifloxacin, respectively. We also found that there were high proportions of MDR *M. tuberculosis* isolates resistant to the fluoroquinolones (Table 3). Approximately 21 % of the isolates were not susceptible to levofloxacin and moxifloxacin. The resistance rates to the 3rd and 4th generation of fluoroquinolones were 15.4–17.3 % and 13.5–15.4 %, respectively. Notably, 13.5 % of the isolates were not susceptible to 5 fluoroquinolones.

**Table 3:** Multiple resistance of MDR-Mtb isolates of 2009 to FQs (*n* = 52)^a^

Category	MIC < 2 mg/L		MIC ≥ 2 mg/L	
	No.	%	No.	%
Lfx + Mfx	29	55.8	11	21.2
Lfx + Mfx + Gfx	24	46.2	8	15.4
Lfx + Mfx + Gmx	27	51.9	9	17.3
Lfx + Mfx + Sfx	26	50.0	8	15.4
Lfx + Mfx + Sfx + Gmx	25	48.1	8	15.4
Lfx + Mfx + Gfx + Gmx	24	46.2	7	13.5
Lfx + Mfx + Gfx + Sfx	24	46.2	7	13.5
Lfx + Mfx + Gfx + Sfx + Gmx	24	46.2	7	13.5

^a^ *MDR* multidrug resistance, *Mtb M. tuberculosis*, *FQs* fluoroquinolones, *MIC* minimum inhibitory concentration, *Lfx* levofloxacin, *Sfx* sparfloxacin, *Mfx* moxifloxacin, *Gmx* gemifloxacin, *Gfx* gatifloxacin

We also analyzed the results of the susceptibility test of *M. tuberculosis* to levofloxacin that was available since 2010. We found that 22.6 % (7/31), 27.6 % (8/29), and 25 % (9/36) MDR isolates from 2010, 2011, and 2012, respectively, were resistant to levofloxacin.

### Factors linked to drug-resistant TB

The risk factors associated with drug resistance were analyzed (Table 4). We found that an age of 35 to 54 (OR 2.5, 95 % CI 1.8–3.4), re-treatment history (OR 5.3, 95 % CI 4.0–7.1), the presence of cavity (OR 1.8, 95 % CI 1.3–2.5), shorter hospitalization (OR 6.4, 95 % CI 4.8–8.7), and infection with the Beijing genotype (OR 4.3, 95 % CI 1.2–15.1) were high-risk factors for the development of MDR-TB based on the univariate analysis. The multivariate analysis further confirmed these findings, except that the Beijing genotype was not included in the final logistic-regression models [26].

**Table 4:** Univariate and multivariate analysis of risk factors for drug-resistant (not MDR) and MDR TB^a^

Characteristic		No. (%) of patients			Drug-resistant vs. Pan-sensitive OR (95 % CI)		MDR vs. Pan-sensitive OR (95 % CI)	
	Total (*n* = 1427)	Pan-sensitive (*n* = 613)	Drug-resistant (*n* = 489)	MDR (*n* = 325)	Univariate analysis	Multivariate analysis	Univariate analysis	Multivariate analysis
Sex								
Male	994(69.7)	425 (69.3)	343(70.1)	226 (69.5)	Referent	NA	Referent	NA
Female	433(30.3)	188 (30.7)	146(29.9)	99 (30.5)	1.0 (0.8–1.3)	NA	1.0 (0.7–1.4)	NA
Age group, y								
7–34	469(32.9)	234(38.2)	152(31.1)	83 (25.5)	Referent	Referent	Referent	Referent
35–54	587(41.1)	198 (32.3)	215(44.0)	174 (53.5)	1.7 (1.3–2.2)	1.5 (1.1–2.0)	2.5 (1. 8–3.4)	1.7 (1.2–2.5)
≥ 55	371(26.0)	181 (29.5)	122(24.9)	68 (20.9)	1.0 (0.8–1.4)	0.9 (0.7–1.2)	1.0 (0.7, 1.5)	0.6 (0.4–0.9)
History of TB treatment								
No	918(64.1)	471 (76.8)	322(65.8)	125 (38.5)	Referent	Referent	Referent	Reference
Yes	509(35.7)	142(23.2)	167(34.2)	200 (61.5)	1.7 (1.3–2.2)	1.7 (1.2–2.1)	5.3 (4.0–7.1)	3.6 (2.6–5.0)
Cavity visible on radiograph^b^								
No	442(35.2)	222 (39.8)	147(34.9)	73 (26.5)	Referent	Referent	Referent	Referent
Yes	812(64.8)	336 (60.2)	274(65.1)	202 (73.5)	1.2 (0.9–1.6)	1.2 (0.9–1.6)	1.8 (1.3–2.5)	1.6 (1.0–2.2)
With standard hospitalization								
Yes	1021(71.5)	496 (80.9)	396 (81.0)	129 (39.7)	Referent	NA	Referent	Referent
No^c^	406(28.5)	117 (19.1)	93(19.0)	196 (60.3)	1.0 (0.7–1.3)	NA	6.4 (4.8–8.7)	5.0 (3.6–7.0)
Beijing genotype^d^								
No	31(10.3)	17 (14.8)	11 (10.2)	3 (3.9)	Referent	NA	Referent	NA
Yes	269(89.7)	98 (85.2)	97 (89.8)	74 (96.1)	1.5 (0.7–3.4)	NA	4.3 (1.2–15.1)	NA

^a^ *MDR* multidrug resistance, *OR* odds ratio, *CI* confidence interval, *NA* not applicable (These variables were not included in the multivariate logistic-regression models)

^b^The radiograph results were available with 1254 patients during the period 2007 through 2012

^c^The hospitalization days of the cases were shorter than the required by the National guidelines: 21–28, 28–35 and 42–56 days for non-MDR new, non-MDR retreated and MDR cases, respectively [26]

^d^Beijing genotype analysis with 300 isolates from 2007 to 2009 was available

Furthermore, MDR-TB cases had a higher frequency (61.5 %) of re-treatment compared to the drug resistant-TB cases (34.2 %). Of the MDR cases, 60.3 % had non-standard hospitalization history compared to 19 % of the drug resistant-TB cases.

Overall, the proportion of cases with cavity, aged 35 to 54, and re-treatment history were 64.8, 41.1, and 35.7 %, respectively, in the TB patients from the study area (Table 4). In this region, the Beijing genotype prevalence was 89.7 %. However, the proportion of cases with non-standard hospitalization was 28.5 %.

## Discussion

Heilongjiang Province is an area of China with high burdens of drug resistant- and MDR-TB [2–4]. However, the reported resistance rates were based on data from the China CDC system. In China, the CDC system generally takes care of outpatients, whereas TB hospitals accept either outpatients or inpatients. The complicated TB cases are usually admitted to TB hospitals. Nationwide studies have shown that first line drug resistance and MDR rates are different among different geographical areas and patient population [1, 10, 27–46] (Table 5). The resistance rates of clinical isolates from 2007 to 2014 to first-line drugs were higher in hospitalized patients [10, 31, 34, 38, 39, 42–44]. On the other hand, surveys based on wide range of TB cases (including outpatients and inpatients) exhibited comparatively lower resistance rates [29, 30, 32, 37]. The MDR prevalence showed similar trend [10, 29–32, 34–46].

**Table 5:** The resistance of Mtb isolated in China to first-line drugs and FQs^a^

Location (Ref.)	Sampling Year	Sampling source^b^	Sample size	First-line Resistance (%)		MDR rate (%)		FQ Resistance (%)^c^	
				Total	New	Total	New	Total	MDR
China [1, 27, 28]	2007	Survey	3929	38.3	34.2	10.2	5.7		
	2007–2008	Survey	3634			9.9		*4.0*	
	2005–2012	Hospital	450	54.4	44.7	25.8	14.5		
Jilin [29]	2008–2011	Survey	1772	40.1	33.1	13.5	8.60		
Ningxia [30]	2013–2014	Survey	665	26.6	20.2	7.8			
Chongqing [31]	2009–2013	Hospital	2271	61.9	52.9	26.3	16.7		
Lianyungang, Jiangsu [32]	2011–2012	Survey	1012	30.4	23.4	8.7	4.2		
Kaohsiung, Taiwan [33]	2000–2008	Hospital	421	15.2		2.1			
Beijing [34]	2007–2009	Hospital	967	70.1	60.9	19.4	14.9	27.1	35.1
Xinjiang [35, 36]	2009–2013	Hospital	410			13.2	12.9		
Heilongjiang (present)	2007–2012	Hospital	1427	57.0	48.7	22.8	13.6		
	2007–2009	Hospital	821	61.9	60.0	26.2	16.5		
	2010–2012	Hospital	606	50.5	41.9	18.2	9.9	10.4	25.0
Kunming, Yunnan [37]	2008–2009	CDC	279	31.5		9.3			
Hunan [38]	2009–2010	Hospital	171	40.9	19.6	25.2	6.2	*10.5*	
Xuzhou, Jiangsu [39]	2011–2012	Hospital	287	16.0		3.14			
Zhejiang [40]	2011–2012	Hospital	1363			16.7			
Rural China [10]	2008	Hospital	380	31.1	25.9	11.3	9.0	10.8	23.3
Jiangxi [41]	2010–2011	Hospital	804			19.8			
Northeast China [42]	2010–2011	Hospital	205	26.3	22.0	6.8	3.0		
Anhui [43]	2010–2011	Hospital	420	29.1	17.1	14.5	6.8	13.3	14.8
Shenzhen [44]	2009	Hospital	589	38.0		9.3		*6.8*	*47.3*
Chongqing [45]	2011–2013	Hospital	1976			10.5			*24.5*
Fujian [46]	2010–2011	Survey	1389			5.4		*2.3*	*25.3*

^a^ *Mtb M. tuberculosis*, *MDR* multidrug resistance, *CDC* Chinese Centre for Disease Control and Prevention, *FQs* fluoroquinolones

^b^Data was derived based on sampling sources of hospital (Hospital), local TB control system (CDC) or nationwide random survey

^c^The data presented the resistance rate of FQ-resistant from total or from MDR Mtb isolates examined (resistant to Ofloxacin, in Italian; resistant to levofloxacin, underlined)

The present investigation included data from a designated TB hospital in Heilongjiang Province from 2007 through 2012, with rates of any first-line drug resistance and MDR-TB of 57.0 and 22.8 %, respectively, whereas these rates were 48.7 and 19.3 %, respectively, in 2012. The rate of drug resistance in this area was highest in 2009 and decreased in 2010 and later. In the investigated hospital, the DST availability was lower than 20 % before 2009, when DST was performed only if patients suffer treatment failure or no obvious effect of treatment. Since 2010, DST became more frequent for culture-positive TB patients. The increased proportion of DST might be the reason for the decrease of drug resistance rates from 2009 to 2010. However, DST is still in only about 50 % of the culture-positive TB cases now. This inadequate DST availability may maintain the comparatively high prevalence of MDR TB.

Primary resistance in this area is also an important issue. Of the new cases investigated in 2012, approximately 40 % were resistant to any first-line drug, whereas 18.5 % were resistant to either isoniazid or rifampin, a population at risk of developing MDR, and 8.9 % were MDR-TB. Notably, approximately 2 % of new cases were resistant to all of the 4 first-line drugs (pan-resistance). This TB population is the potential resource of the XDR-TB if the treatment strategy is not appropriate. Regarding China’s TB treatment regulations [26, 48], all new pulmonary TB cases are treated free of charge with first-line drugs if DST is not available.

Furthermore, less than 20 % of the TB medical facilities that use second-line drugs for MDR-TB treatment design therapy regimens based on DST [34]. This situation will cause the appearance of additional MDR and XDR *M. tuberculosis* cases.

In the new cases, high proportions with cavity (approximately 60 %) and short hospitalization (19 %) should also be identified. These patients are released from hospitals after the initial treatment and may be a highly dangerous source of infection for susceptible individuals, causing the spread of both TB and MDR TB. It is important to decrease the proportion of non-standard hospitalization and appropriately treat the cases with cavity to achieve the successful control of primary TB spread.

The resistance situation to FQs distributed variously in different TB populations (outpatients and inpatients) and in different areas of China, with a higher resistance rate in hospitalized patients. In addition, the MDR strains would be more likely to resistant to FQs [10, 34, 38, 43–47].

In recent years, levofloxacin, the most frequently used fluoroquinolone in China, has become the first choice fluoroquinolone for MDR-TB treatment, and moxifloxacin is used as an alternative fluoroquinolone based on China’s clinical practice guidelines for TB [26].

Based on the present data, there is a limited choice of fluoroquinolones for the ideal regimen for MDR-TB therapy because approximately 15–17 % and 14–15 % of the MDR-TB cases are not susceptible to the 3 and 4 of fluoroquinolones, respectively. Importantly, at least 13 % of the MDR *M. tuberculosis* isolates were not susceptible to any of the 5 fluoroquinolones. The high prevalence of drug resistance may become uncontrolled if measures are not effective and may indicate an increase of XDR-TB.

MDR is an independent risk factor associated with resistance to fluoroquinolones [9]. The high rate of MDR-TB in the investigated area (22.8 %) is a related causative reason for the high prevalence of fluoroquinolone resistance. Furthermore, the fluoroquinolones are commonly used antibiotics for clinical departments, and antibiotics abuse has not been well controlled in China [11–14]. Controlling the resistance to fluoroquinolones may be achieved through the proper management of MDR-TB cases and the proper use of such antibiotics.

## Conclusions

The results of our study indicate that there was a high prevalence of drug resistance to the first-line drugs and multidrug resistance (MDR) in Heilongjiang Province, northeastern China. Among MDR TB, more than 10 % was resistant to fluoroquinolones, indicating a severe second line drug resistance. After analysis of the risks of MDR-TB, we should pay more attention to patients’ age, re-treatment proportion, cavity lesion, and high proportion of shorter hospitalization to get control tuberculosis more efficiently.

## Abbreviations

DOTS: Directly observed treatment short course
DST: Drug susceptibility testing
MDR: Multidrug resistant
MGIT: Mycobacterium Growth Indicator Tube
MICs: Minimum inhibition concentrations
TB: Tuberculosis
WHO: World Health Organization
XDR: Extensively drug-resistant
